# Ectopic Pituitary Adenoma Presenting as a Clival Mass

**DOI:** 10.7759/cureus.4158

**Published:** 2019-02-28

**Authors:** Juan J Altafulla, Joshua T Prickett, Graham Dupont, R. Shane Tubbs, Zachary Litvack

**Affiliations:** 1 Neurosurgery, Seattle Science Foundation, Seattle, USA; 2 Neurosurgery, Swedish Neuroscience Institute, Seattle, USA

**Keywords:** pituitary adenoma, clival mass, histology

## Abstract

Pituitary adenomas are well described in the literature and are frequently observed and treated in clinical practice by neurosurgeons. On the other hand, ectopic adenomas are a diagnostic enigma; a good understanding of anatomy and radiological characteristics is crucial for the successful management of such pathologies. In this paper, we describe the case of a 77-year-old woman who presented with a clival mass invading the left cavernous sinus; we also discuss the associated diagnostic techniques, approaches, imaging options, and characteristics.

## Introduction

Understanding the normal anatomy of the skull base, in particular, the sellar and parasellar regions, is fundamental for diagnosing and treating pathologies associated with such landmarks. The sellar and parasellar regions are in direct relation with the sphenoid bone. The sella turcica, a midline depression in the posterior sphenoid bone, limited anteriorly by the tuberculum sellae and the anterior clinoid processes, posteriorly by dorsum sellae and the posterior clinoid processes, laterally by the cavernous sinus, and the pituitary gland comprised by its anterior lobe or adenohypophysis and posterior lobe or neurohypophysis are the main structures forming the sellar region [1].

The parasellar region is formed by the cavernous sinus, a trabeculated venous channel that extends from the superior orbital fissure to the petrous apex. Cranial nerves III, IV, V_1_, and V_2_ are located in the lateral aspect while the carotid and abducens nerve travel more medially. Also contributing to the parasellar region are the suprasellar cistern structures, the hypothalamus (most ventral portion of the diencephalon) lying anterioinferiorly to the thalamus and comprising the floor and portions of the walls of the third ventricle. It is bounded anteriorly by the optic chiasm, laterally by optic nerves, and posteriorly by the mammillary bodies. The infundibulum extends off of the hypothalamus between the optic chiasm and the tuber cinereum. The hypothalamic-pituitary network is primarily supplied by the superior hypophyseal artery, a branch from the internal carotid. This arterial branch contains fenestrated capillaries and the blood-brain barrier is absent. The porosity of this vessel permits direct secretion of the aforementioned hormones into the bloodstream [1-2].

## Case presentation

**Figure 1:**
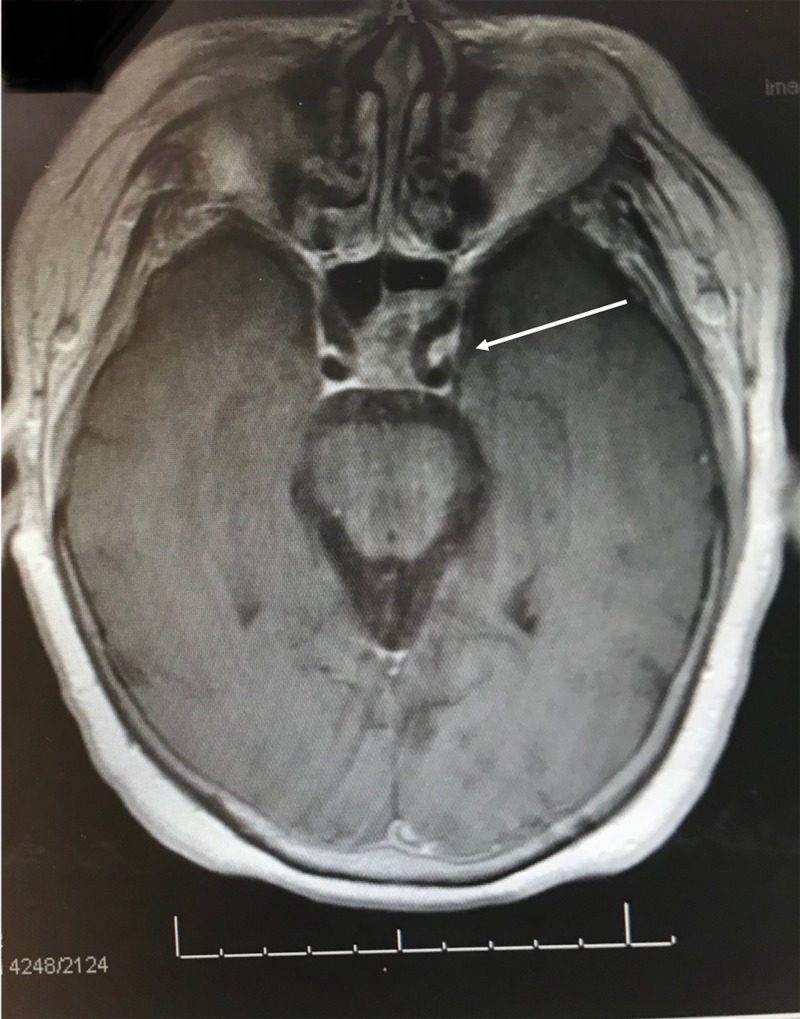
T1 post-contrast axial magnetic resonance imaging (MRI) showing heterogenous contrast enhancement of a clival mass that invades the left cavernous sinus, encasing the internal carotid artery

**Figure 2:**
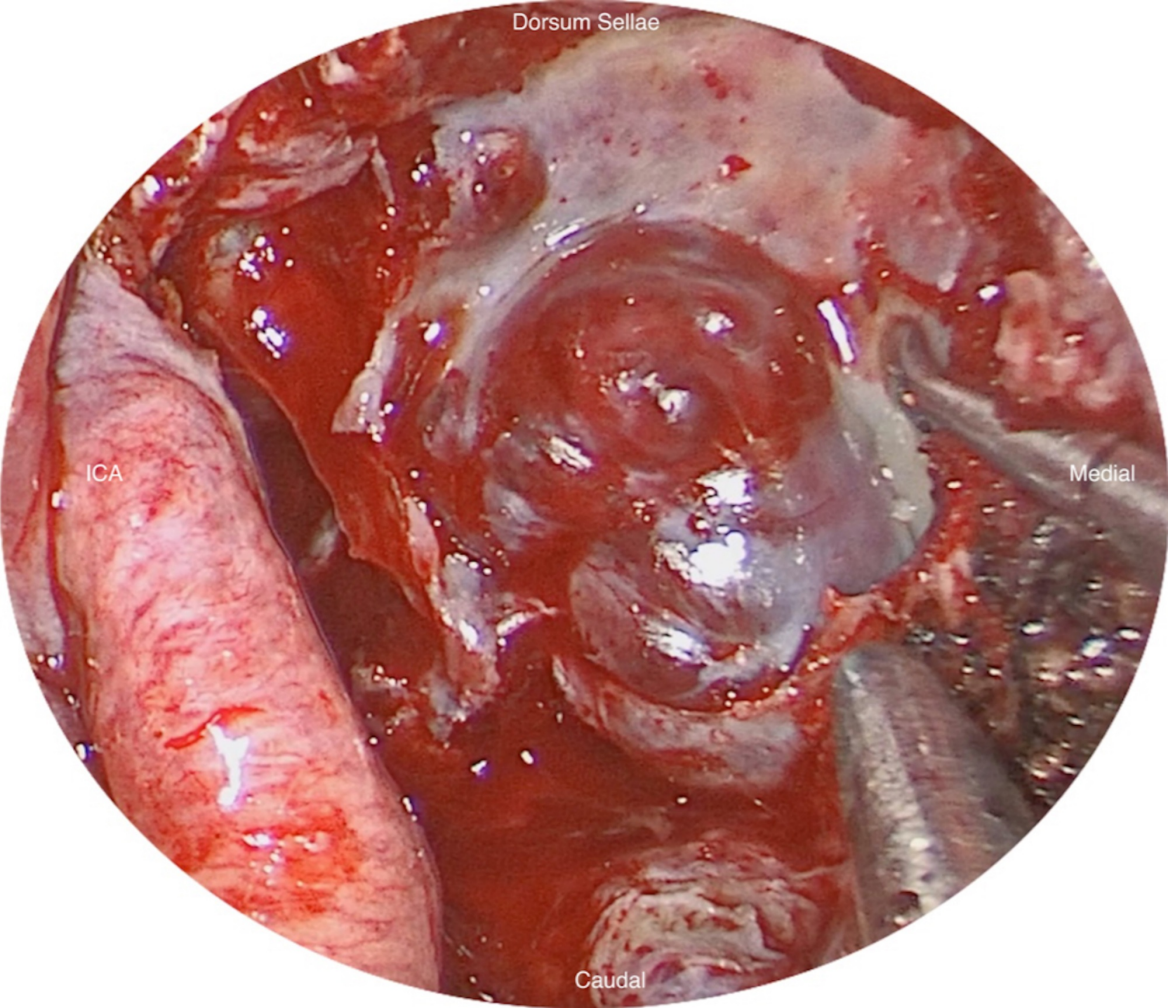
Endoscopic view of the clival mass after dissection from the internal carotid artery (ICA)

**Figure 3:**
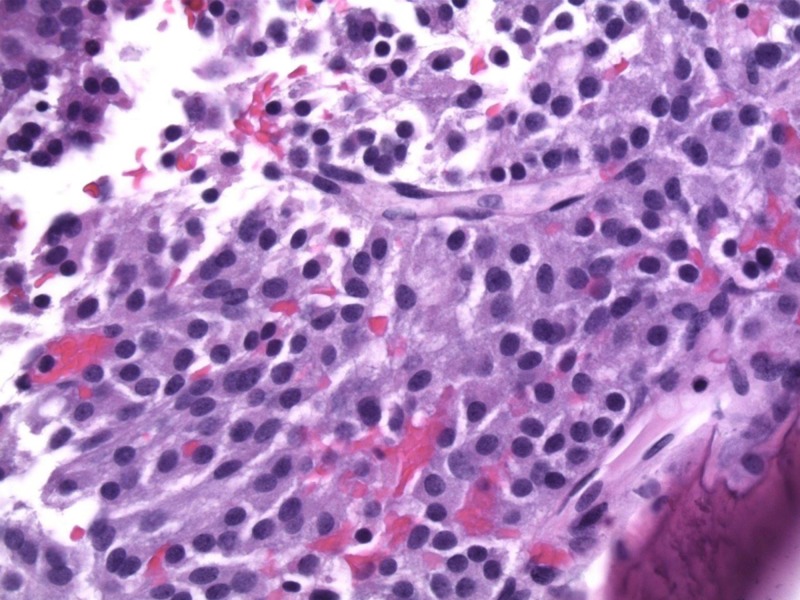
Hematoxylin and eosin (H&E) stain of the clival tumor

A 77-year-old female presented with a left clival mass, which was found incidentally on magnetic resonance imaging (MRI) (Figure 1). Neurological examination was normal, except casual headaches. Past medical history includes functional endoscopic sinus surgery (FESS) and septoplasty in 1998, and ongoing chronic sinus issues. A positron emission tomography-computed tomography scan (PET-CT) was performed to exclude primary neoplasm. The patient was admitted for biopsy and resection of left clival mass. The patient underwent a total resection of the mass via transsphenoidal endoscopy; the mass was found to be isolated from the sellar and suprasellar area, with no connection between the mass and the pituitary gland nor stalk, and was found on a different plane in the clivus (Figure 2). The patient was discharged three days later with no neurological deficit. The histopathology was submitted as “ectopic pituitary adenoma, null cell type” (Figure 3).

## Discussion

Due to its embryological and histological diversity, the sellar and parasellar region imaging is still somewhat challenging. The pituitary gland is a small-volume organ with an average height of 5.4 ± 0.9 mm and should never exceed 10 mm, with the exception of physiologic hypertrophy that occurs during pregnancy and lactancy with the hypophysis measuring up to 12 mm [3]. Two main theories regarding the origin of ectopic pituitary adenomas are proposed in the literature. The first theory being that the adenomas occurring in the sphenoidal sinus, nasal cavity, or suprasellar region, are thought to arise from the embryological remnants of pituitary tissue since it occurs alongside structures associated with the embryological development of Rathke’s pouch; the second theory proposes that adenomas presenting over the suprasellar region may also arise from anterior lobe cells that are attached to the supradiaphragmatic portion of the pituitary stalk [4]. Computed tomography (CT) is crucial to evaluate bony structures invaded by tumor and calcifications within, or the anatomy of the sphenoidal sinus in order to plan for an extended endonasal approach. Conventional radiography has lost its importance in such settings. MRI is the study of choice for evaluating the sellar and parasellar regions. Standard MRI protocol includes conventional and dynamic imaging, thin section sagittal and coronal T1-weighted images (T1WI) with and without contrast enhancement, thin sectional T2-weighted images (T2WI), and a whole brain scan with T2 or Flair-weighted imaging [5].

The adenohypophysis comprises almost 70% of the total size of the gland in newborns and up to three or four months of age. It may exhibit some hyperintensity which will gradually disappear, and as early as six months, should exhibit isointensity with the adjacent brain [6]. The posterior lobe can be easily identified in healthy patients and infants. In T1-weighted MRI, it appears as an area of increased signal pre-contrast, due to high content of antidiuretic hormone secretory granules. It is not seen in patients with inappropriate antidiuretic hormone secretion syndrome. According to Al-Dahmani et al., adenomas account for 83% of sellar masses while non-pituitary lesions make up the remaining 17%. Overall female preponderance is 68% with a mean age of presentation of 44.6 +-18 years old [7]. Ectopic pituitary adenoma is rare and may arise from the nasopharynx, clivus, sphenoidal sinus, cavernous sinus, third ventricle, or the suprasellar region. All the reported cases seem to present with a hormonal hypersecretion syndrome, including hyperprolactinemia, Cushing’s disease, acromegaly, and hyperthyroidism. MRI will always show an isointense mass in comparison to gray matter on T1WI and enhances heterogeneously with the application of contrast [8]. Its clinical presentation ranges from severe to mild disease and will ultimately depend on the quantity and type of hormone secreted, as well as the location of the tumor (pressure effect on surrounding structures).

Clinical presentation can be classified into three categories: endocrinological, neurological, and ophthalmological. Endocrinological presentations are related to the hormones secreted by the gland and mostly present as hypersecretory syndromes. For instance, hyperprolactinemia may produce decreased libido, impotence, and gynecomastia in men; while in women, it presents typically with amenorrhea and galactorrhea [9]. Ophthalmological presentations are directly proportionate to the tumor location and size. Adenomas arising from the diaphragm sellae into the suprasellar cistern will produce a bitemporal hemianopsia secondary to compression of the inferonasal fibers from below. The superotemporal fields are affected first, the progress continues inferiorly and medially, and the superonasal field is affected last. In contrast, bitemporal hemianoptic scotomas (with or without inferior field defects) indicate the involvement of the posterior chiasm notch where there is a higher concentration of macular crossing fibers [10]. Neurologically, headache is the most common symptom presenting in up to 76% of cases [11], often due to compression of pain-sensitive structures located in the internal carotid artery (ICA), trigeminal nerve and ganglion, or cavernous sinus dura (lateral and medial walls). Headache may also arise as a result of raised intracranial pressure due to obstruction of cerebral spinal fluid, primarily through the third ventricle. Upward extension toward the hypothalamus may result in hypothalamic obesity caused by disruption of appetite control [9].

The sphenoidal sinus is the most common site for ectopic pituitary adenomas, and must be differentiated from other lesions that can originate from this area such as chordomas, which present as a destructive lesion showing variable signal intensity on both T1WI and T2WI. When contrast enhancement is low, infectious agents such as fungus will be observed in an MRI with intermediate signal on T1WI and low signal intensity on T2WI [12]. On MRI, sphenoidal sinus ectopic pituitary adenomas usually present isointense relative to adjacent grey matter, and show mild to moderate enhancement after the administration of contrast material. They are usually heterogeneous, demonstrating foci of low signal intensity on T1WI and high signal intensity on T2WI [12]. Viera Jr. et al. used the Knosp-Steiner classification for parasellar tumor extension and concluded that about 10% of pituitary adenomas invade the cavernous sinus, in which case, the encasement of the intracavernous ICA greater than 45% is considered the most specific sign of invasion (specificity of 93.5% and sensitivity of 92.1%), and when encasement was <25%, the cavernous sinus was never invaded [13]. They also added that the cavernous sinus was invaded when three or more venous compartments, or the lateral venous compartment, were not visible. Invasion is discarded when the medial venous compartment is intact or a normal gland interface is seen between the adenoma and the cavernous sinus [14].

For treatment options, surgical resection via the endoscopic binostril endonasal remains the main pillar as it offers a greater angle of attack and greater surgical freedom than the endoscopic uninostril endonasal, or the endoscopic contralateral transmaxillary approach for accessing parasellar structures. Nonetheless, the endoscopic contralateral transmaxillary approach provides a more lateral exposure of the ipsilateral parasellar area, and a shorter surgical trajectory to the contralateral parasellar area [15].

## Conclusions

Ectopic pituitary adenomas are a rare finding. Imaging plays a big role in diagnosis and treatment. MRI is preferably used to determine tumor localization and extension, whereas CT scan aids in the resection planning and approach selection. Depending on the aforementioned characteristics exhibited by the tumor, a surgical approach is selected. The endoscopic endonasal has gained much popularity mainly for its straight-forward access, and good and rapid recovery with minimal cosmetic defects.
